# Neuropathological correlations of ^18^F-florzolotau PET in a case with Pick’s disease

**DOI:** 10.1186/s13550-025-01296-6

**Published:** 2025-07-31

**Authors:** Hisaomi Suzuki, Manabu Kubota, Shin Kurose, Kenji Tagai, Hironobu Endo, Mitsumoto Onaya, Yasuharu Yamamoto, Naruhiko Sahara, Masahiro Ohgidani, Chie Haga, Hiroya Hara, Haruhiko Akiyama, Keisuke Takahata, Makoto Higuchi

**Affiliations:** 1 Advanced Neuroimaging Center, Institute for Quantum Medical Science, National Institutes for Quantum Science and Technology, 4- 9-1 Anagawa, Inage, Chiba 263-8555 Japan; 2 Department of Neuropsychiatry, Keio University School of Medicine, 35 Shinanomachi, Shinjuku, Tokyo, 160-8582 Japan; 3 Department of Psychiatry, National Hospital Organization Shimofusa Psychiatric Center, 578 Heta-cho, Midori-ku, Chiba, 266-0007 Japan; 4 Department of Psychiatry, Kyoto University Graduate School of Medicine, 54 Shogoin Kawahara-cho, Sakyo-ku, Kyoto, 606-8507 Japan; 5 Department of Clinical Laboratory, National Center of Neurology and Psychiatry, 4-1-1 Ogawahigashi, Kodaira, Tokyo, 187-8551 Japan; 6 Department of Psychiatry, Jikei University Graduate School of Medicine, 3-19-18 Nishi-Shinbashi, Minato-ku, Tokyo, 105-8461 Japan; 7 Department of Neuroscience and Pathobiology, Research Institute of Environmental Medicine, Nagoya University, Furo-cho, Chikusa-ku, Nagoya, Aichi, 464-8601 Japan; 8 Department of Functional Anatomy and Neuroscience, Asahikawa Medical University, 2-1-1-1 Midorigaoka Higashi, Asahikawa, Hokkaido, 078-8510 Japan; 9 Dementia Research Project, Tokyo Metropolitan Institute of Medical Science, 2-1-6 Kamikitazawa, Setagaya-ku, Tokyo, 156-8506 Japan; 10 Neuroetiology and Diagnostic Science, Osaka Metropolitan University Graduate School of Medicine, 1-4-3 Asahimachi, Abeno-ku, Osaka, 545-8585 Japan

**Keywords:** Florzolotau, Frontotemporal lobar degeneration, Neuropathology, Pick’s disease, Positron emission tomography, Tauopathy, Three repeat tau

## Abstract

**Background:**

Pick’s disease (PiD) is classified as frontotemporal lobar degeneration with pathological tau aggregates. Positron emission tomography (PET) with ^18^F-florzolotau provides high-contrast imaging of diverse tau fibrils. While our previous work demonstrated the detectability of three repeat (3R) tau pathology by ^18^F-florzolotau PET in an autopsy-confirmed PiD patient, its potential for quantitative assessment of 3R tau aggregates in living individuals remains unclear. In this study, we analyzed correlations between in vivo ^18^F-florzolotau retentions and postmortem neuropathological data across brain regions in the same case with PiD.

**Case presentation:**

The patient was 60 years of age at the time of death and had been diagnosed with behavioral variant frontotemporal dementia. The patient underwent ^18^F-florzolotau PET one year prior to death and was given the pathological diagnosis of PiD by brain autopsy. Regional tau pathology was assessed using Bodian’s silver staining and immunohistochemistry with a monoclonal antibody (AT8). Histopathological assays revealed abundant intraneuronal Pick bodies along with neuropil threads in frontotemporal and other brain areas. In the cerebral cortex, AT8-positive areas exhibited a significant positive correlation with ^18^F-florzolotau binding in the corresponding regions (Pearson’s *r* = 0.81, *p* < 0.001) estimated as standardized uptake value ratio corrected for partial volume effect. In contrast, no such associations were found in subcortical structures. Furthermore, a substantial proportion of Pick bodies displayed fluorescence co-labelled with florzolotau and AT8 antibodies.

**Conclusions:**

Collectively, the present findings support the capability of ^18^F-florzolotau PET for the in vivo quantification of 3R tau fibrils.

**Supplementary Information:**

The online version contains supplementary material available at 10.1186/s13550-025-01296-6.

## Background

Pick’s disease (PiD) was originally described by Arnold Pick as a disease characterized by progressive behavioral and language disturbances and focal atrophy of the frontotemporal lobes [1]. Its diagnostic criteria have been revised over time, and currently, cases with pathological Pick bodies are classified as PiD. From a pathological standpoint, PiD is classified as frontotemporal lobar degeneration with tau aggregates (FTLD-tau) [2]. The primary clinical manifestation of PiD is behavioral variant frontotemporal dementia (bvFTD), a major FTD phenotype associated with alterations in personality, social behavior, emotion, and insight [3]. However, PiD can also manifest as semantic dementia, progressive non-fluent aphasia, and, less commonly, corticobasal syndrome [4]. As such, substantial clinicopathologic heterogeneity exists between these clinical manifestations and their associated underlying neuropathological features [5].

We previously developed a tau PET radioligand of ^18^F-florzolotau (also referred to as florzolotau (18F), ^18^F-APN-1607, or ^18^F-PM-PBB3) [6]. This ligand enables high-contrast imaging of diverse tau fibrils characteristic of both Alzheimer’s disease (AD) and non-AD tauopathies, including three repeat (3R) tau fibrils exemplified by PiD and four repeat (4R) tau fibrils observed in corticobasal degeneration (CBD) and progressive supranuclear palsy (PSP). Our in vitro assay and in vivo human PET imaging studies demonstrated ^18^F-florzolotau’s ability to detect a wide range of tau aggregates, including 3R and 4R isoforms. However, to date no imaging-neuropathological correlations have been reported in 3R tauopathy brains, using ^18^F-florzolotau. Validating ^18^F-florzolotau’s capacity to detect and quantify 3R pathology through imaging-pathological evaluations in the same individual with PiD pathology could contribute to future diagnostic and therapeutic approaches for the disease.

Therefore, in this study we utilized PET imaging with ^18^F-florzolotau to investigate correlations between PET radioligand accumulations and postmortem neuropathological assessments in a patient diagnosed with PiD.

### Case presentation

This study was approved by the Radiation Drug Safety Committee and the Institutional Review Board of the National Institutes for Quantum Science and Technology, Japan, and the Institutional Ethical Review Board of the Shimofusa Psychiatric Medical Center, Japan. This study was conducted in accordance with the Code of Ethics of the World Medical Association. After a complete description of the study, written informed consent was obtained from the patient’s legal representative. In addition, written informed consent was obtained from the legal representative of the patient for the publication of this case report. Parts of the patient’s data are included in our previous research [6, 7].

The patient, a right-handed man, was 60 years old at the time of his death. His developmental history revealed no particular issues. Around age 54, he experienced interpersonal troubles at work and at home, leading to deviant social behavior. His first psychiatric visit was at 54 years of age. His initial score on the revised Hasegawa’s Dementia Scale (HDS-R) [8] was 23 out of 30 points, above the cut-off point of 20–21 for possible dementia. His brain MRI image at that time revealed temporal lobe atrophy centered on the hippocampal gyrus with hemispheric differences and frontal lobe atrophy centered on the orbital surface.

During his hospital visits, he became irritable, ate at several restaurants a day, and ate cemetery offerings at night. Due to worsening disinhibition and socially deviant behaviors, he was admitted to the hospital with a diagnosis of probable FTD [9]. He displayed shallow and euphoric responses, and FTD-characteristic behaviors such as fixed-path wandering. With chlorpromazine of 50 mg/day and sodium valproate of 400 mg/day, his euphoria lessened, but indifference and lack of motivation increased, leading to discontinuation of sodium valproate.

After discharge, he was referred to the National Hospital Organization Shimofusa Psychiatric Medical Center at age 57 due to worsening deviant behavior and wandering, and was admitted twice. As the disease progressed, his activity level decreased, contractures developed, speech disappeared, and he barely responded to calls.

**Fig. 1:**
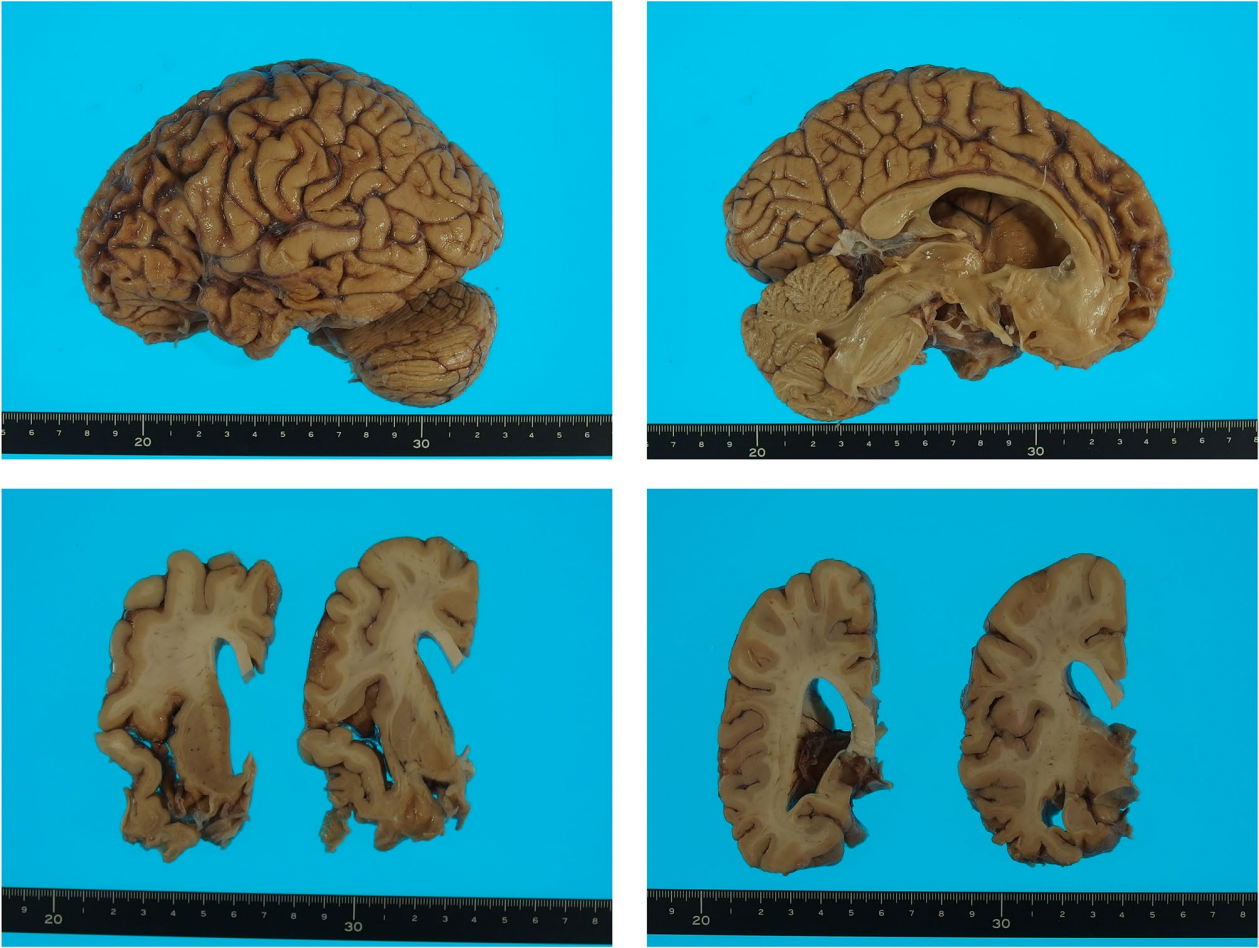
Macroscopic images of the brain. Lateral, medial, and coronal slices of the brain showing severe atrophy in the frontotemporal cortices and ventricular enlargement. Atrophy was not evident in the parietooccipital cortices or in the cerebellum

At the age of 59 years, PET scans with ^11^C-PiB and ^18^F-florzolotau were performed to quantify amyloid-beta (Aβ) and tau accumulations, in addition to an MRI scan. His severe clinical symptoms made detailed neurological evaluation challenging; however, neurological examination at the time of the PET scans showed no obvious amyotrophy or pyramidal signs, while forced grasp and Myerson’s sign were present. His Mini-Mental State Examination (MMSE) and Frontal Assessment Battery (FAB) scores were zero. The Neuropsychiatric Inventory (NPI) score was 54, indicating apathy, disinhibition, aberrant motor behavior, nightmare behavior disturbances, and appetite and eating disturbances. The Stereotypy Rating Inventory (SRI) [10] score was eight, indicating the presence of stereotypic movement behaviors. The following year, he suffered from recurrent aspiration pneumonia, and he died of pneumonia and pyothorax, one year after the PET scans. Brain autopsy was performed at the National Hospital Organization Shimofusa Psychiatric Center. Macroscopic images of the brain are shown in Fig. 1.

### PET and MRI acquisition and data processing

Radiosynthesis of ^11^C-PiB and ^18^F-florzolotau was carried out as described elsewhere [11, 12]. PET scans of ^11^C-PiB and ^18^F-florzolotau were conducted with ECAT Exact HR + and mCT flow systems, respectively. A T1-weighted MR image was obtained using a 3-T MRI scanner (MAGNETOM Verio, Siemens, Germany). Details of the scan parameters for these scan devices are described elsewhere [7].

For ^11^C-PiB PET, 50 min after an intravenous rapid bolus injection of 529 MBq of the radioligand, a 20-min PET acquisition (4 × 5-min frames) was performed. For ^18^F-florzolotau, 90 min after an intravenous rapid bolus injection of 183 MBq of the radioligand in a dim room to avoid photoracemisation, 20-min PET acquisition (4 × 5-min frames) was performed.

The PET images were corrected for scatter using the single-scatter simulation method. A head fixation device was used to minimize the subject’s head movement during PET measurements. Motion-corrected PET images were co-registered to the corresponding T1-weighted MR images using the PMOD^®^ software ver. 3.8 (PMOD Technologies Ltd., Zurich, Switzerland). Parametric PET images were generated by voxel-based calculation of the standardized uptake value ratio (SUVR) to the cerebellar GM (excluding the vermis) for ^11^C-PiB and ^18^F-florzolotau, respectively.

Surface-based cortical reconstruction and volumetric subcortical segmentation of the MR image were performed with FreeSurfer software (version 6.0.0; http://surfer.nmr.harvard.edu). Regions of interest (ROIs) were defined using the cerebral atlases and maps provided as defaults in FreeSurfer [13–16] for the subsequent analyses. For the ^18^F-florzolotau PET data in these ROIs, we applied partial volume correction (PVC) by the volume-of-interest-based Geometric Transfer Matrix method [17].

### Neuroimaging findings

**Fig. 2:**
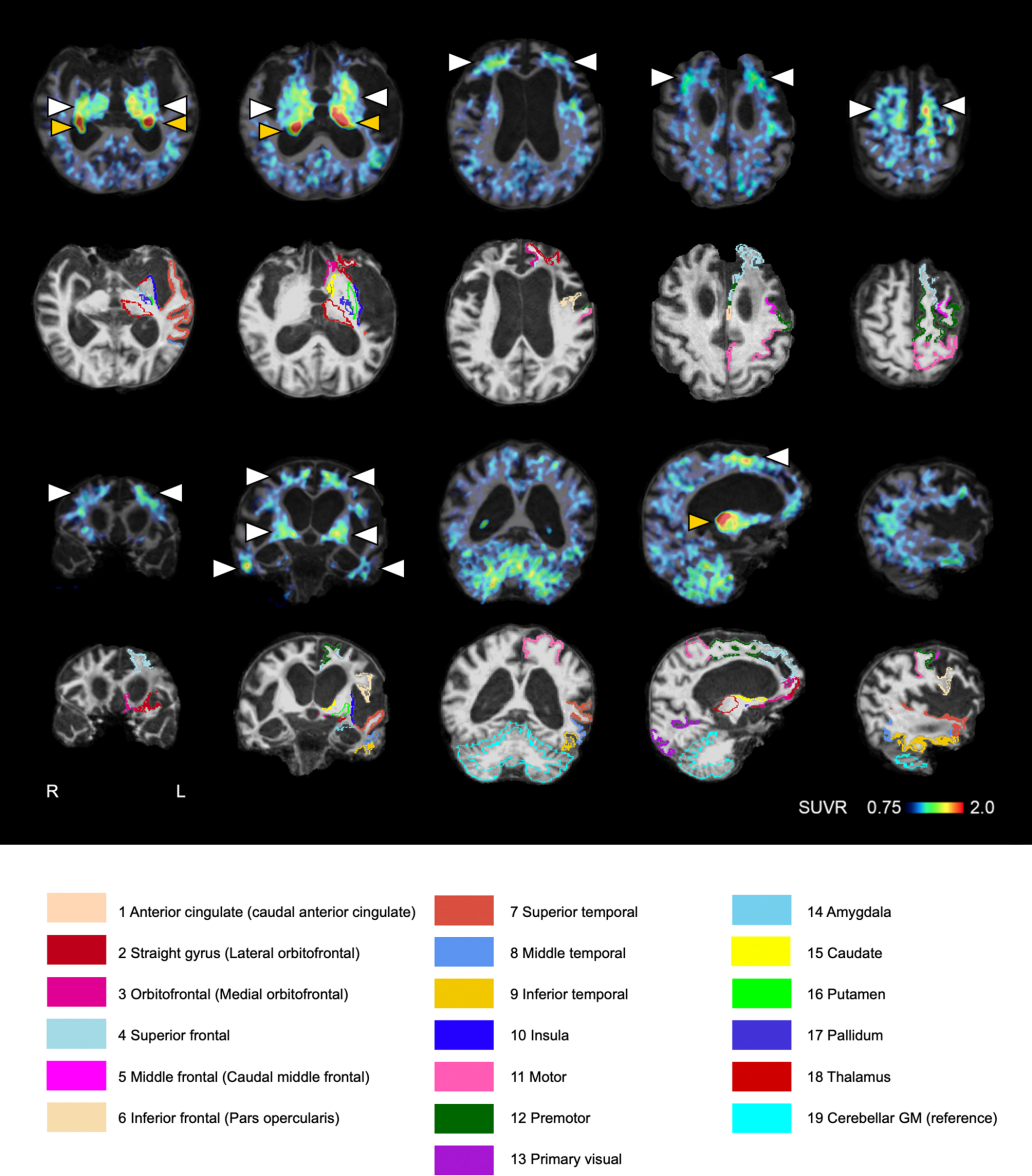
Images of ^18^F-florzolotau PET SUVR, along with T1-weighted MRI and ROIs. SUVR Images of ^18^F-florzolotau PET are shown along with T1-weighted MRI images. White arrowheads indicate enhanced parenchymal radioligand retention, and yellow arrowheads denote radioactivity accumulations in the choroid plexus supposedly unrelated to tau depositions. ROIs except for the cerebellar gray matter reference are for the left hemisphere only. Only the outermost boundary of each ROI is outlined. Abbreviations: ROI, region of interest; SUVR, standardized uptake value ratio

MRI demonstrated severe regional atrophy in the frontotemporal cortices, medial temporal regions, and basal ganglia, as well as marked ventricular enlargement (Fig. 2). The SUVR images of ^11^C-PiB and ^18^F-florzolotau PET without PVC were visually examined. The ^11^C-PiB SUVR image did not indicate the presence of Aβ deposition according to the standard method of visual assessment by three researchers [18]. The ^18^F-florzolotau SUVR image showed elevated radioligand retention in the inferior, middle, and superior frontal cortex, inferior and middle temporal cortex, and basal ganglia (Fig. 2). The PET signal was also found in the choroid plexus, which is thought to be an off-target signal. The accumulations in the cerebral cortex were right-hemisphere dominant.

### Neuropathological examination

An autopsy was performed four hours after death. The brain weight was 1170 g. Macroscopically, severe atrophy was observed in the frontotemporal cortices of the brain (Fig. 1). The brain was fixed with 20% formalin and examined after approximately one month. Tissues from the superior, middle, and inferior frontal cortices, orbitofrontal cortex, superior, middle, and inferior temporal cortices, anterior cingulate cortex, insular cortex, straight gyrus, motor and premotor cortices, and primary visual cortex, as well as the caudate, putamen, pallidum, thalamus, and amygdala were prepared. These tissue samples were embedded in paraffin and sectioned at a thickness of 6 μm for hematoxylin–eosin (HE) staining and 9 μm for the other staining methods. The latter included the Klüver–Barrera (KB), Bodian, methenamine silver and Gallyas–Braak methods as well as immunohistochemical staining.

For immunostaining, deparaffinized tissues were pretreated with formic acid for antibody to Aβ and autoclave treatment (10 mM sodium citrate buffer, pH 6.0, at 120 °C for 10 min) for other antibodies. To eliminate endogenous peroxidase activity, tissues were incubated with 0.5% H_2_O_2_ in 0.01 M phosphate buffered saline (PBS) for 30 min. After washing three times with 0.01 M PBS, pH 7.4, tissues were blocked with 10% normal serum from the appropriate animal species. They were then incubated overnight at 4 °C with one of the primary antibodies in PBS containing 0.3% Triton X-100 (PBS-Tx). Following three washes in PBS-Tx, the tissues were incubated in a secondary antibody (EnVision + Dual Link System-HRP, Agilent Technologies, California, USA) to rabbit and mouse immunoglobulins conjugated with HRP-labeled polymers for 2 h. Peroxidase labeling was visualized using 0.2% 3,3′-diaminobenzidine (DAB) as a chromogen, and the tissues were counterstained with hematoxylin.

Immunohistochemistry was performed using primary antibodies against phosphorylated tau (Phospho-Tau (Ser202, Thr205) Monoclonal Antibody (AT8), mouse, monoclonal, 1:1000, Thermo Fisher Scientific, Massachusetts, USA), phosphorylated TAR DNA binding protein-43 (TDP-43) (Anti TDP-43 phosphorylated at Ser409 and Ser410, rabbit, polyclonal, 1:1000, Cosmo Bio, Tokyo, Japan), phosphorylated α-synuclein (#1175, rabbit, polyclonal, 1:1000 [19]), and Aβ (E50 Anti-Aβ17–31, rabbit, polyclonal, 1:1000).

The stained sections were scanned and the microscopic images were imported as digital photo files using the NanoZoomer Digital Pathology (NDP) system (Hamamatsu Photonics, Hamamatsu, Japan). Cropped regions were generated using the NDP view at a magnification of ×200 and 18 independent areas, including 13 cortical and five subcortical regions. The number of pixels in the DAB positive was calculated using MATLAB with the Image Processing Toolbox 2023a (MathWorks).

### Neuropathological findings

**Fig. 3:**
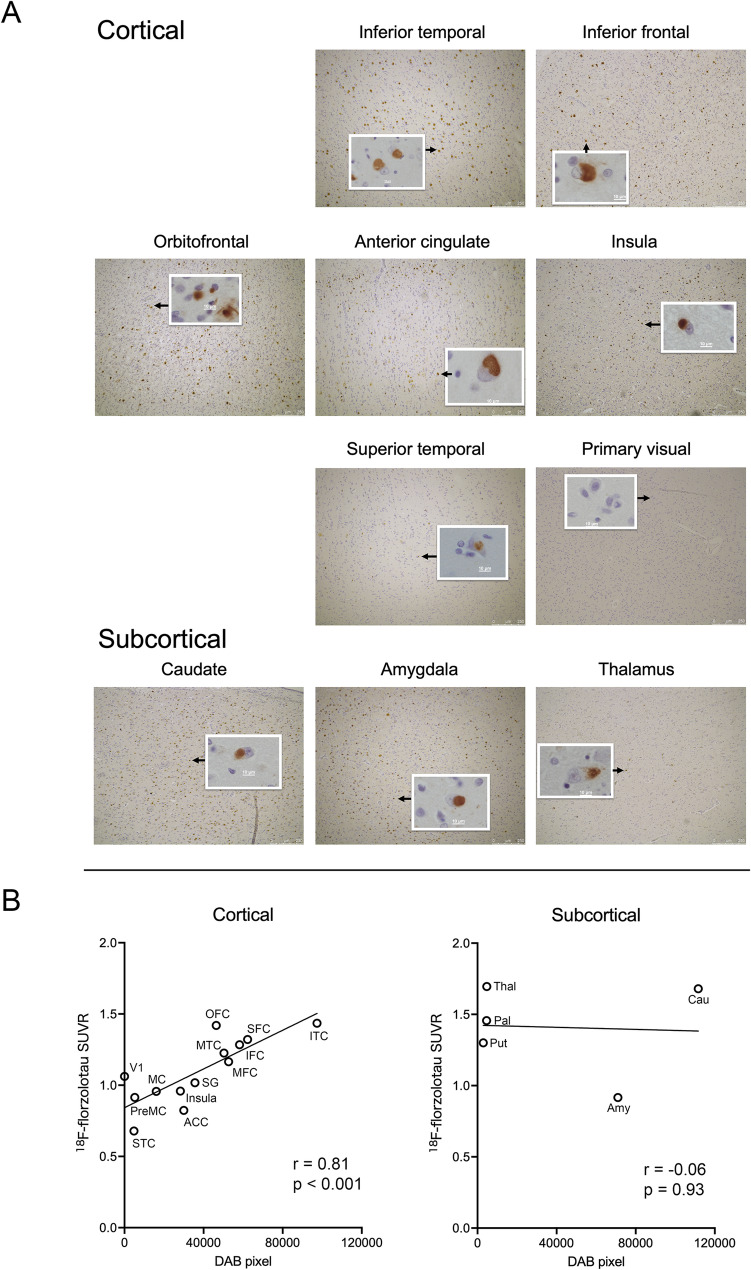
AT8 immunohistochemistry of tau pathology and correlation with ^18^F-florzolotau PET. (**A**) Representative images illustrating regional tau pathology in a case of Pick’s disease using AT8 immunohistochemistry. (**B**) Scatter plots showing correlations between partial volume-corrected ^18^F-florzolotau SUVR and the AT8-positive area (number of DAB-positive pixels) in various ROIs, shown separately for cortical and subcortical regions. Abbreviations: ACC, anterior cingulate cortex; Amy, amygdala; Cau, caudate; DAB, 3,3′-diaminobenzidine; IFC, inferior frontal cortex; ITC, inferior temporal cortex; MC, motor cortex; MFC, middle frontal cortex; MTC, middle temporal cortex; OFC, orbitofrontal cortex; Pal, pallidum; PET, positron emission tomography; PreMC, premotor cortex; put, putamen; r, Pearson’s r; ROI, region of interest; SFC, superior frontal cortex; SG, straight gyrus; STC, superior temporal cortex; Thal, thalamus; V1, primary visual cortex; SUVR, standardized uptake value ratio

Neuropathological examination of the brain confirmed the diagnosis of PiD with Pick bodies. Both Bodian’s silver staining and immunohistochemistry with AT8 revealed the occurrence of numerous Pick bodies. The Pick bodies were Gallyas staining-negative. As shown in Fig. 3AB, the density of the Pick bodies was highest in the caudate nucleus and inferior temporal cortex, followed by the amygdala, superior, inferior, and middle frontal cortices, middle temporal cortex, orbitofrontal cortex, straight gyrus, anterior cingulate gyrus, and insular cortex. Pick bodies were also found, but less frequently, in motor and premotor cortices, thalamus, pallidum, putamen, and superior temporal cortex. Pick bodies were not detected in the primary visual cortex. Such a distribution of Pick bodies corresponds to phase 3 of the staging by Irwin et al. [20]. Neither neurofibrillary tangles nor neuropil threads were observed in the entorhinal cortex or in other brain regions examined in this study. Aβ immunohistochemistry and methenamine silver staining showed the absence of Aβ deposition. Immunohistochemistry for phosphorylated α-synuclein demonstrated only a small number of positive inclusions scattered in the temporal neocortex, with no significant spread that could be considered clinically relevant. No phosphorylated TDP-43 positive structure was present. Furthermore, no significant glial pathology, including aging-related tau astrogliopathy (ARTAG), was observed.

### Correlations between ^18^F-florzolotau PET SUVR values and postmortem Tau pathology assessed by AT8-positive areas

Correlational analysis was performed to examine associations between ^18^F-florzolotau SUVR values with PVC and the total tau burden as determined by pixels with positive DAB staining in each ROI, for cortical and subcortical regions separately. Statistical analysis was conducted using SPSS (version 27.0; SPSS Inc.). The statistical significance threshold was set at *p* < 0.05 (two-tailed).

For cortical regions, a significant positive correlation was found between ^18^F-florzolotau SUVR values and the number of DAB-positive pixels (Pearson’s *r* = 0.81, *p* < 0.001; Fig. 3B). On the other hand, no significant correlation was found for the subcortical regions.

To assess the impact of PVC, we conducted a similar correlational analysis between ^18^F-florzolotau SUVR prior to applying the PVC and the number of DAB-positive pixels. A significant correlation was found for the cortical regions only, albeit with a slightly less robust statistical value (*r* = 0.72, *p* = 0.006; Supplementary Figure S1).

### Double Florzolotau fluorescence and phospho-tau antibody Immunofluorescence labelling

**Fig. 4:**
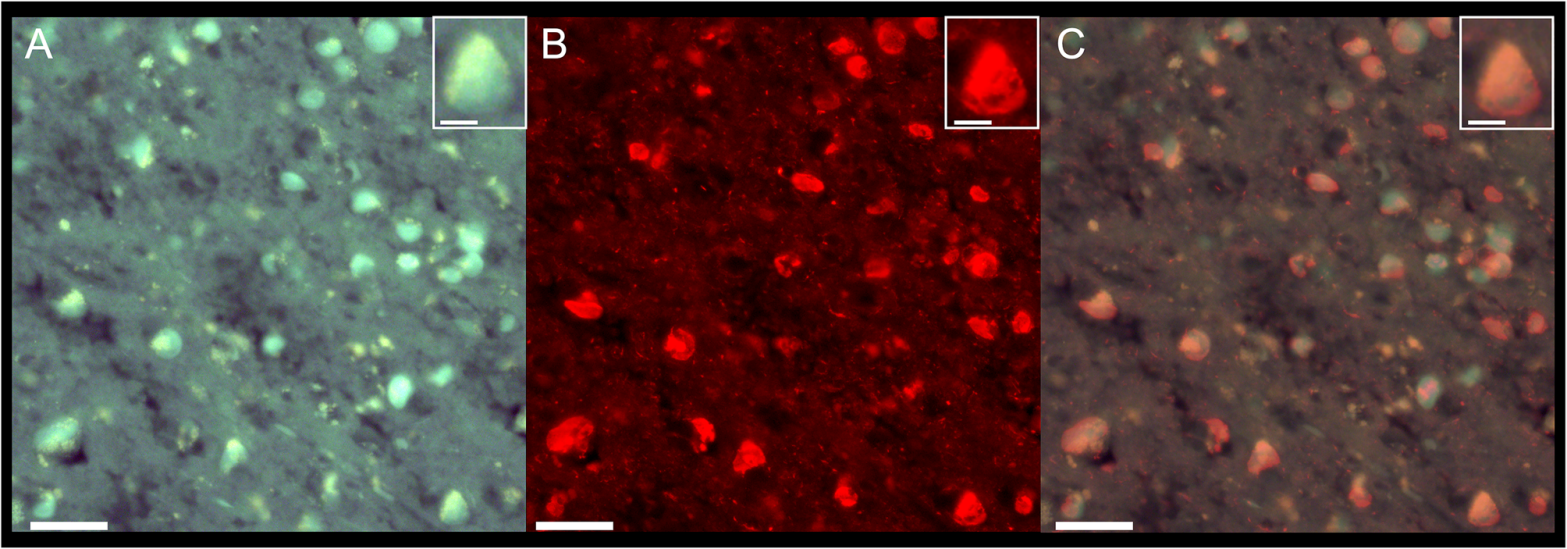
Florzolotau and phosphorylated tau antibody-positive inclusions in the inferior temporal gyrus. (**A–C**) The same sections were stained with florzolotau and phospho-tau antibody. Histochemical labelling florzolotau (**A**) and immunohistochemical staining with phospho-tau antibody (AT8) (**B**) were performed on sections of the inferior temporal gyrus. (**A**) A number of Pick bodies labelled with florzolotau. (**B**) A number of Pick bodies labelled with AT8. (**C**) A merged image of A and B shows labelling of Pick bodies with both florzolotau and AT8. The insets in A–C show a Pick body that was positive for both florzolotau and AT8. Scale bar in A–C = 50 μm; insets = 10 μm

To confirm ^18^F-florzolotau binding to tau deposits in PiD, brain sections of the inferior temporal gyrus were labelled using florzolotau fluorescence and phospho-tau antibody immunofluorescence. Deparaffinized sections were incubated in non-radiolabelled florzolotau (25 mM) with 50% ethanol for 30 min at room temperature, followed by washing with 50% ethanol for 5 min, dipped in distilled water twice for 3 min each, and mounted on non-fluorescent mounting media (VECTASHIELD, Vector Laboratories). Fluorescence images were captured using a DM4000 microscope (Leica) equipped with a custom filter cube for PBB3 (excitation bandpass at 414/46 nm and suppression low-pass with a 458 nm cutoff) [21]. After capturing the images, sections underwent 95% formic acid treatment for 30 min. For antigen retrieval, paraffin-sections were incubated in citric acid solution [0.01 M sodium citrate, 0.01 M citric acid (pH 6.0)] at 90℃ for 30 min, followed by blocking with a solution containing 4% BSA, 2% horse serum, and 0.25% Triton X-100 in PBS. After PBS washes, sections were incubated with primary antibodies AT8 (1:500), followed by fluorescently conjugated secondary antibodies (1:500, Jackson ImmunoResearch). A considerable proportion of Pick bodies in the inferior temporal gyrus were double-labelled with florzolotau and AT8 antibody (Fig. 4).

## Discussion

This is the first study of neuroimaging-pathological correlations in a patient with PiD, combining ^18^F-florzolotau PET and postmortem immunochemical techniques. The patient presented with a clinical manifestation of bvFTD. Our analysis revealed a significant positive correlation between ^18^F-florzolotau PET signals and Pick body density, as quantified by AT8 immunochemistry, in neocortical regions. Additionally, double staining in the inferior temporal gyrus showed that a substantial proportion of Pick bodies were co-labelled with florzolotau and AT8 antibodies.

Several PET radioligands, including ^18^F-flortaucipir (^18^F-AV1451), ^18^F-MK6240, ^18^F-THK5351, and ^18^F-THK5317, have been investigated for visualizing tau aggregates in vivo. While these radioligands can detect neuronal somatodendritic tau inclusions composed of all six isoforms observed in AD, in vivo detection of 3R tau isoforms has been challenging due to lower radioligand signal strength, off-target binding, or a lack of postmortem validation for non-AD aggregates [22, 23]. A previous ^18^F-flortaucipir PET study that included a PiD case reported that, although AT8 immunohistochemistry revealed a high tau burden in the temporal and parietal lobes of the case, ¹⁸F-flortaucipir uptake remained relatively uniform across brain regions, suggesting its limited sensitivity to 3R tau [24]. Although ^18^F-PI-2620 has shown potential for detecting pathologies associated with PSP and CBD in addition to AD [25], its efficacy in identifying 3R tauopathies has yet to be established. In contrast, we previously demonstrated that ^18^F-florzolotau effectively detects various tau pathologies both in vitro and in vivo [6]. Moreover, a recent ^18^F-florzolotau PET study showed that patients with clinical diagnosis of FTD can be classified into putative neuropathology-based subgroups (3R-like, 4R-like, AD-like, and tau-negative) based on visual and quantitative PET assessments [7].

In the present case, the patient’s duration of illness was five years at the time of the PET scan, and severe cognitive impairment, significant language impairment, and motor dysfunctions were observed, indicating an advanced stage of bvFTD. Our postmortem neuropathological examination confirmed the phase 3 disease progression, with Pick bodies observed in the motor cortex [20], aligning with the patient’s clinical presentation. The neocortical PET radioligand accumulation was most prominent in the frontotemporal regions, particularly the orbital and inferior frontal, superior frontal, and inferior temporal cortices. This distribution strongly correlated with the regional tau pathology observed immunohistochemically in the postmortem brain (Fig. 3B). Furthermore, our double staining analysis confirmed that a substantial proportion of Pick bodies were co-labelled with florzolotau and AT8 antibody, supporting the high accuracy of ^18^F-florzolotau in detecting and quantifying PiD pathology.

Conversely, no significant correlation was identified between ^18^F-florzolotau accumulation and neuropathological findings in subcortical regions. One explanation may be the effect of nonspecific accumulation in certain subcortical areas, such as the basal ganglia or thalamus [26]. Although prior studies have confirmed that florzolotau does not bind to either MAO-A or MAO-B [6], the possibility of nonspecific binding or interaction with unidentified substances in subcortical regions cannot be dismissed. However, our previous study demonstrated a higher ^18^F-florzolotau accumulation in the caudate of this patient compared to controls ([7]; this case is presented as “P01”). Therefore, the signal in this region likely reflects pathological alterations rather than nonspecific accumulation observed in both patients and cognitively healthy individuals. Another plausible explanation for the absence of the correlation in the subcortical regions is that, despite the implementation of PVC, heterogeneous subcortical atrophy may influence the results. Indeed, in bvFTD, previous studies have suggested that subcortical atrophy precedes focal cortical atrophy, with more severe atrophy in the caudate and accumbens [27]. Given this, the differential atrophy between the caudate and putamen observed in this case [7] may have impacted the results. Furthermore, there is a possibility that ¹⁸F-florzolotau accumulation in subcortical regions reflects its cross-reactivity with transmembrane protein 106B (TMEM106B) aggregates [28]. However, florzolotau fluorescence labeling of brain sections did not reveal any florzolotau-positive signals with the characteristic short filamentous morphology suggestive of TMEM106B aggregation, in regions such as the putamen and thalamus. Finally, it should be noted that the SUVR in the thalamus may be partially influenced by spill-over from the choroid plexus, as illustrated in Fig. 2. However, this spill-over effect was not observed in any other ROIs. Therefore, the SUVR value in the thalamus of this particular case should be interpreted with considerable caution. We accordingly conducted correlational analyses between ^18^F-florzolotau SUVR and the number of DAB-positive pixels in the subcortical regions exclusive of the thalamus (SUVR with PVC, *r* = 0.17, *p* = 0.83; SUVR without PVC, *r* = -0.79, *p* = 0.21), and confirmed that the results did not exhibit significant changes.

Concerning the impact of brain atrophy and PVC on ^18^F-florzolotau SUVR, while the cortical ^18^F-florzolotau SUVR without PVC demonstrated a significant correlation with the number of DAB-positive pixels similar to the correlation observed with the PVC-applied SUVR, it exhibited less statistical robustness. These findings suggest that focal brain atrophy partially obscures the potential association between tau radioligand accumulation and postmortem tau burden. Notably, in both cortical and subcortical regions, SUVR values increased following PVC, particularly in areas with pronounced atrophy, such as the caudate, and prefrontal, and middle and inferior temporal cortical regions (see Supplementary Figure S2). Consequently, SUVRs without PVC tend to be underestimated in regions of severe focal atrophy, whereas SUVRs with PVC maintain a clearer correlation with postmortem tau pathology assessed by the number of DAB-positive pixels.

The strengths of this study include the use of the tau PET ligand ^18^F-florzolotau, which provides high-contrast imaging of diverse tau lesions, as well as access to well-preserved brain specimens with only a four-hour postmortem delay. However, several limitations should be taken into account. First, severe brain atrophy presented challenges in the neuroimaging-pathological correlation analysis. While PVC was applied to mitigate this issue, it may not fully account for the atrophy-related effects on our analysis. Second, the approximately one-year interval between PET scans and autopsy may complicate interpretation of the results, although this duration aligns with previous neuroimaging-neuropathological studies [29]. Third, the potential for the radioligand to bind to unknown non-specific substances needs to be investigated in future studies. Fourth, although we investigated the correlation between florzolotau PET signals and the AT8-positive area, as well as their co-localization in a patient with PiD, it is important to acknowledge that these findings do not necessarily indicate that florzolotau specifically binds to Pick bodies in PiD. Lastly, as this study is based on a single case, future analyses involving multiple patients with varying clinical presentations will be needed to generalize the current findings.

## Conclusions

In this neuroimaging-pathological examination of a PiD case, we demonstrated a significant positive correlation in the neocortical regions between ^18^F-florzolotau PET-measured tau accumulation and Pick pathology quantified by AT8 immunochemistry, although no clear correlation was found for the subcortical regions. Double staining in the inferior temporal gyrus revealed that a substantial proportion of Pick bodies were co-labelled with florzolotau and AT8 antibodies. The present findings provide evidence supporting ^18^F-florzolotau PET as a useful tool for in vivo quantification of 3R tauopathy, contributing to future molecular-targeted treatment of neurodegenerative dementia based on putative neuropathology.

## Supplementary Information

Below is the link to the electronic supplementary material.

Supplementary Material 1

## Data Availability

The data that support the findings of this study are available from the corresponding author upon reasonable request.

## Abbreviations

AD: Alzheimer’s disease
ARTAG: aging-related tau astrogliopathy
Aβ: amyloid-beta
bvFTD: behavioral variant frontotemporal dementia
CBD: corticobasal degeneration
DAB: 3,3′-diaminobenzidine
FAB: Frontal Assessment Battery
FTD: frontotemporal dementia
FTLD: frontotemporal lobar degeneration
FTLD-tau: frontotemporal lobar degeneration with tau aggregates
GM: gray matter
HDS-R: revised Hasegawa’s Dementia Scale
HE: hematoxylin–eosin
KB: Klüver–Barrera
MMSE: Mini-Mental State Examination
NDP: NanoZoomer Digital Pathology
NPI: Neuropsychiatric Inventory
PBS: phosphate buffered saline
PET: positron emission tomography
PiD: Pick’s disease
PSP: progressive supranuclear palsy
PVC: partial volume correction
ROI: region of interest
SRI: Stereotypy Rating Inventory
SUVR: standardized uptake value ratio
TDP-43: TAR DNA binding protein-43
TMEM106B: transmembrane protein 106B
3R: three repeat
4R: four repeat
