# Supplementary material for: Neuropathological correlations of 18F-florzolotau PET in a case with Pick’s disease
Source: EJNMMI Res. 2025 Jul 31;15:96. doi: 10.1186/s13550-025-01296-6 (PMC12314175; doi:10.1186/s13550-025-01296-6)
Supplement: Supplementary file 1 — Supplementary Material 1 [file 13550_2025_1296_MOESM1_ESM.pdf]

Title page

Title:

Neuropathological correlations of ^{18}F-florzolotau PET in a case with Pick's disease

Authors:

Hisaomi Suzuki[1,2,3]*, Manabu Kubota[1,4]*, Shin Kurose[1,2,5], Kenji Tagai[1,6], Hironobu Endo[1], Mitsumoto Onaya[2,3], Yasuharu Yamamoto[1,2], Naruhiko Sahara[1,7], Masahiro Ohgidani[8], Chie Haga[9], Hiroya Hara[9], Haruhiko Akiyama[9], Keisuke Takahata[1,2], Makoto Higuchi[1,10]

1 Advanced Neuroimaging Center, Institute for Quantum Medical Science, National Institutes for Quantum Science and Technology, 4-9-1 Anagawa, Inage, Chiba, Chiba 263-8555, Japan

2 Department of Neuropsychiatry, Keio University School of Medicine, 35 Shinanomachi, Shinjuku, Tokyo 160-8582, Japan

3 Department of Psychiatry, National Hospital Organization Shimofusa Psychiatric Center, Chiba, 578 Heta-cho, Midori-ku, Chiba 266-0007, Japan

4 Department of Psychiatry, Kyoto University Graduate School of Medicine, 54 Shogoin Kawahara-cho, Sakyo-ku Kyoto 606-8507, Japan

5 Department of Clinical Laboratory, National Center of Neurology and Psychiatry, 4-1-1 Ogawahigashi, Kodaira, Tokyo 187-8551, Japan

6 Department of Psychiatry, Jikei University Graduate School of Medicine, 3-19-18 Nishi-Shinbashi, Minato-ku, Tokyo 105-8461, Japan

7 Department of Neuroscience and Pathobiology, Research Institute of Environmental Medicine, Nagoya University, Furo-cho, Chikusa-ku, Nagoya, Aichi 464-8601 Japan

8 Department of Functional Anatomy and Neuroscience, 2-1-1-1 Midorigaoka Higashi, Asahikawa, Hokkaido 078-8510, Japan

9 Dementia Research Project, Tokyo Metropolitan Institute of Medical Science, 2-1-6 Kamikitazawa, Setagaya-ku, Tokyo 156-8506, Japan

10 Neuroetiology and Diagnostic Science, Osaka Metropolitan University Graduate School of Medicine, 1-4-3 Asahimachi, Abeno-ku, Osaka 545-8585, Japan

* These authors contributed equally.

Corresponding author:

Manabu Kubota, M.D., Ph.D.

Advanced Neuroimaging Center, Institute for Quantum Medical Science, National Institutes for Quantum Science and Technology

Address: 4-9-1 Anagawa, Inage-ku, Chiba, Chiba 263-8555, Japan Tel. +81-43-206-3251

Fax. +81-43-253-0396

Email: kubota.manabu@qst.go.jp, m_kubota@kuhp.kyoto-u.ac.jp

Supplementary Information

Supplementary Figure S1

Scatter plots showing correlations between ^{18}F-florzolotau SUVR without PVC and the AT8-positive area (number of DAB-positive pixels) in various ROIs, shown separately for cortical and subcortical regions.

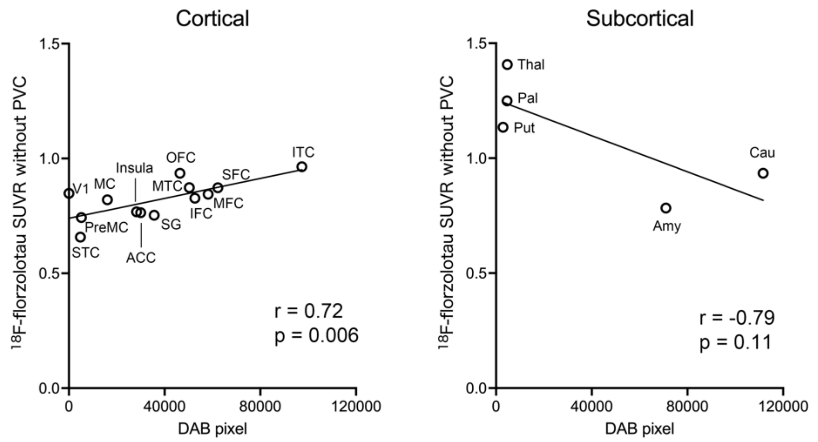

Abbreviations: ACC, anterior cingulate cortex; Amy, amygdala; Cau, caudate; DAB, 3,3′ - diaminobenzidine; IFC, inferior frontal cortex; ITC, inferior temporal cortex; MC, motor cortex; MFC, middle frontal cortex; MTC, middle temporal cortex; OFC, orbitofrontal cortex; Pal, pallidum; PET, positron emission tomography; PreMC, premotor cortex; put, putamen; PVC, partial volume correction; r, Pearson's r; ROI, region of interest; SFC, superior frontal cortex; SG, straight gyrus; STC, superior temporal cortex; Thal, thalamus; V1, primary visual cortex; SUVR, standardized uptake value ratio

Supplementary Figure S2

Scatter plots showing correlations between ^{18}F-florzolotau SUVR and volumes in various ROIs, shown separately for cortical and subcortical regions.

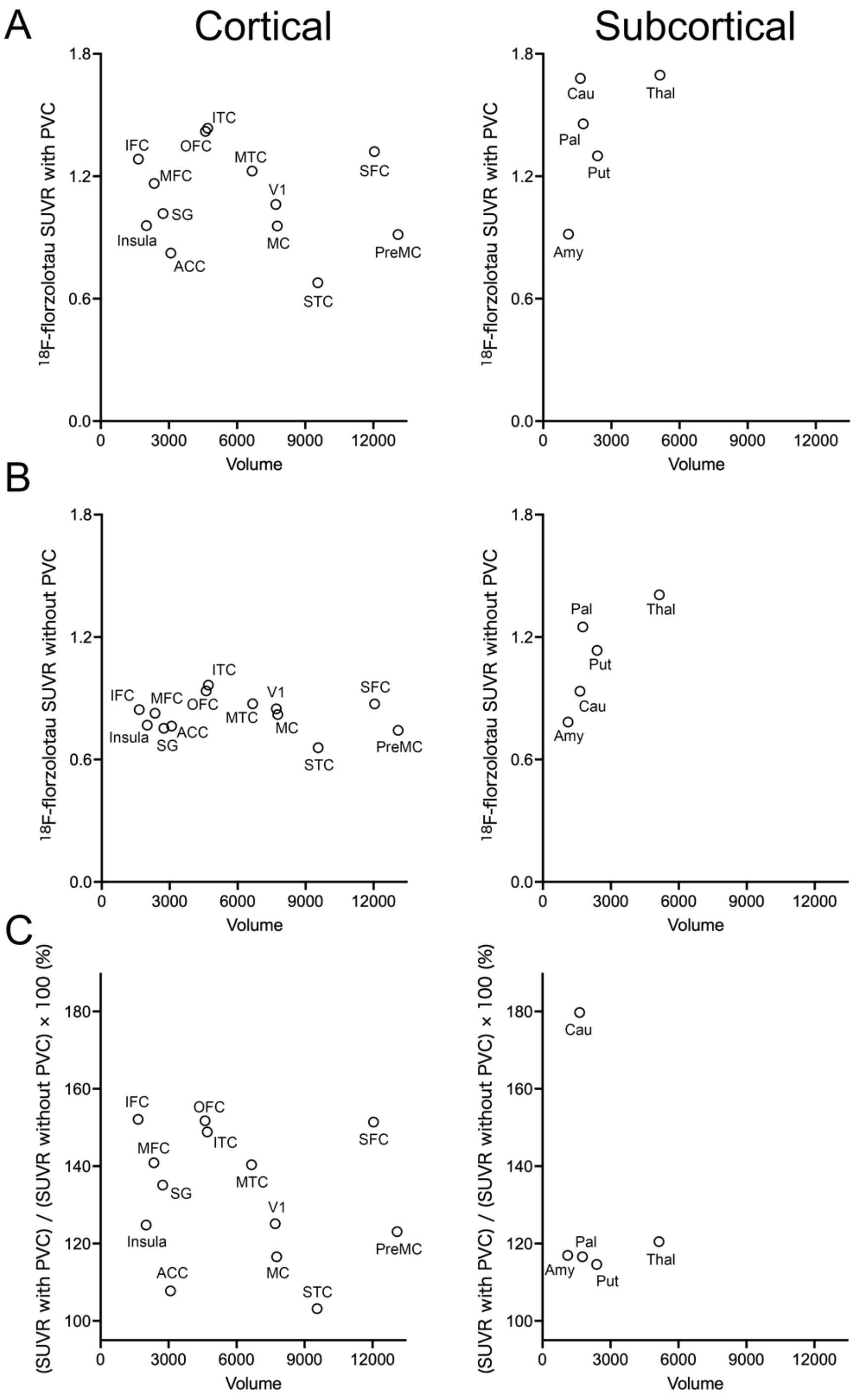

Correlations for ^{18}F-florzolotau SUVR with PVC (A), without PVC (B), and for the ratio of SUVR values with PVC to those without PVC (C).

Abbreviations: ACC, anterior cingulate cortex; Amy, amygdala; Cau, caudate; DAB, 3,3′-diaminobenzidine; IFC, inferior frontal cortex; ITC, inferior temporal cortex; MC, motor cortex; MFC, middle frontal cortex; MTC, middle temporal cortex; OFC, orbitofrontal cortex; Pal, pallidum; PET, positron emission tomography; PreMC, premotor cortex; put, putamen; PVC, partial volume correction; r, Pearson's r; ROI, region of interest; SFC, superior frontal cortex; SG, straight gyrus; STC, superior temporal cortex; Thal, thalamus; V1, primary visual cortex; SUVR, standardized uptake value ratio
